# Traumatic Testicular Dislocation Associated with Lateral Compression Pelvic Ring Injury and T-Shaped Acetabulum Fracture

**DOI:** 10.1155/2016/9706392

**Published:** 2016-09-08

**Authors:** Daniel Howard Wiznia, Mike Wang, Chang Yeon-Kim, Paul Tomaszewski, Michael P. Leslie

**Affiliations:** Department of Orthopaedics and Rehabilitation, Yale University School of Medicine, 800 Howard Avenue, New Haven, CT 06510, USA

## Abstract

We report a case of a unilateral testicular dislocation to the superficial inguinal region associated with a lateral compression type pelvic ring injury (OTA classification 61-C3.3a2, b2, c3) and left T-shaped acetabulum fracture (OTA classification 62-B2) in a 44-year-old male who was in a motorcycle accident. The testicular dislocation was noted during the emergency department primary survey, and its location and viability were verified with ultrasound. The testicle was isolated during surgical stabilization of the left acetabulum through a Pfannenstiel incision and modified-Stoppa approach and returned through the inguinal canal to the scrotum. In follow-up, the patient did not suffer urologic or sexual dysfunction. All motorcycle collision patients presenting with pelvic ring injuries or acetabulum fractures should be worked up for possible testicular dislocation with a scrotal exam. Advanced imaging and a urologic consult may be necessary to detect and treat these injuries.

## 1. Introduction

Traumatic testicular dislocation is a rare finding most frequently found as part of a spectrum of anterior posterior compression type pelvic ring fractures associated with motorcycle collisions [1–5]. The following report describes a testicular dislocation with a lateral compression type pelvic ring injury and a T-type acetabulum fracture, which is unique to the literature. The purpose of this report is to raise awareness of the potential association of lateral compression type pelvic ring injuries and testicular dislocations in the context of motorcycle-related trauma, as well as describe comanagement of this presentation. Knowledge of this presentation will likely prevent iatrogenic injury and the associated comorbidities of an unrecognized testicular dislocation. We have obtained the patient's written informed consent for print and electronic publication of this report.

## 2. Case Report

**Figure 1:**
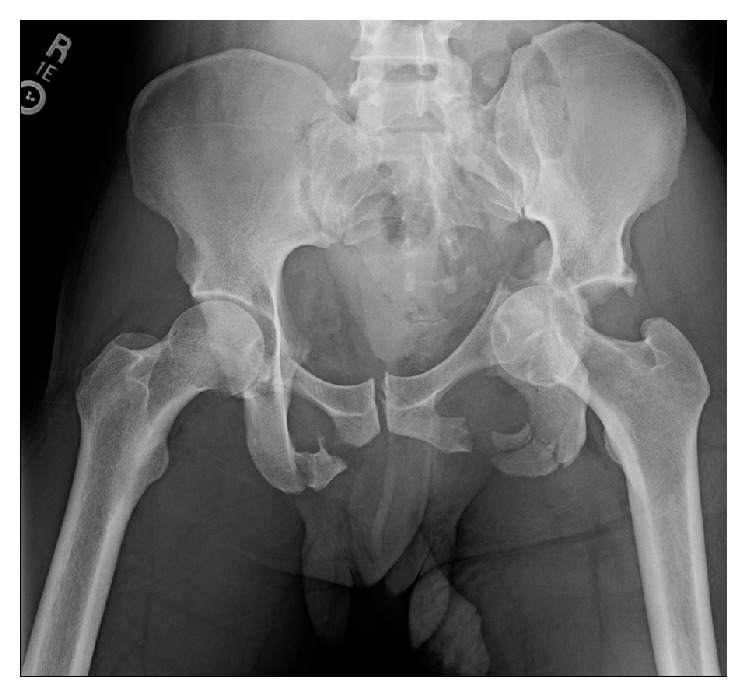
Trauma series AP pelvis radiograph.

**Figure 2:**
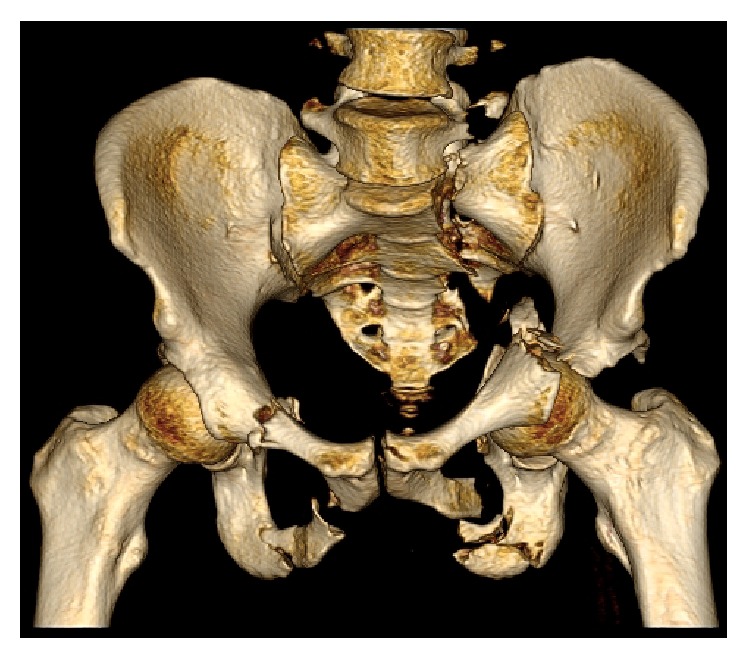
3D pelvis preoperative CT reconstruction.

**Figure 3:**
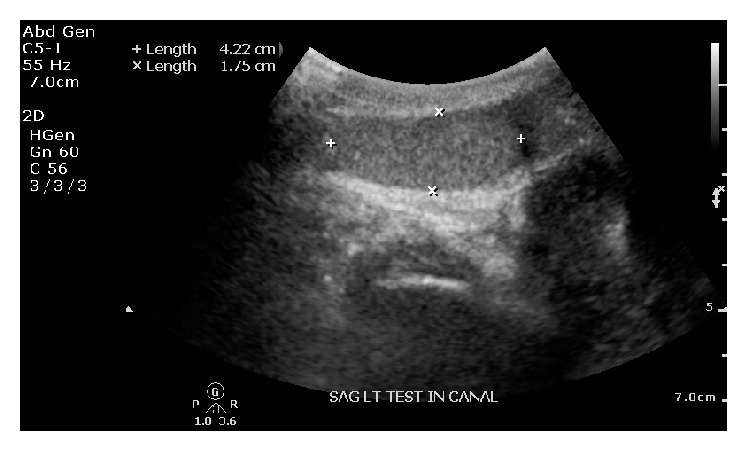
Ultrasound of left testicle in the inguinal canal.

A 44-year-old nonhelmeted motorcycle rider presented to the emergency department after suffering a front end collision. Clinical exam demonstrated a Glasgow Coma Scale of 14. His right arm was positioned overhead with the shoulder in full abduction and elbow in flexion, his left leg was shortened with a foot drop, and only one testicle was palpable in the scrotum. Imaging demonstrated a lateral compression pelvic ring injury which included a type II dens left-sided sacral fracture with dissociation and bilateral superior and inferior pubic rami fractures (OTA classification 61-C3.3a2, b2, c3) [6]. In addition, there was a left-sided T-shaped acetabulum fracture (OTA classification 62-B2) [6] with protrusio and right shoulder luxatio erecta (Figures 1 and 2). Ultrasound demonstrated that the left testicle was in the inguinal canal with normal Doppler wave forms (Figure 3). Urology was unable to relocate the testicle with external pressure.

**Figure 4:**
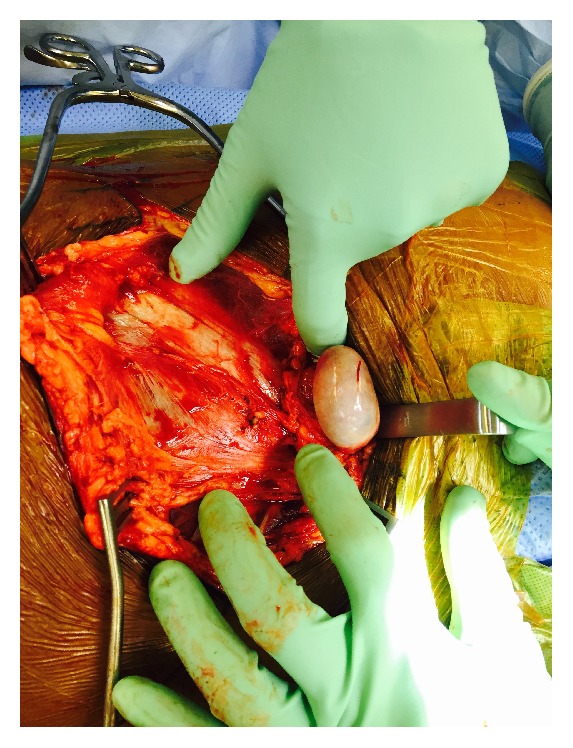
Intraoperative photograph of left testicle within the wound superior to the fascia overlying the inguinal canal.

On hospital day three, the patient went to the operating room for an open reduction and internal fixation of the left acetabular fracture. The posterior column of the acetabulum was addressed via a Kocher Langenbeck approach [7] and the anterior column was addressed with a Pfannenstiel incision and a modified-Stoppa approach [8]. As dissection was carried down to the level of the rectus fascia, the inguinal canal was noted to be completely disrupted and the left testicle was noted to be within the wound superior to the fascia overlying the inguinal canal (Figure 4). Soft tissue trauma suggested that the testicle was ejected through the superficial ring. Urology assisted in returning the testicle to the scrotum and confirmed blood flow with ultrasound.

**Figure 5:**
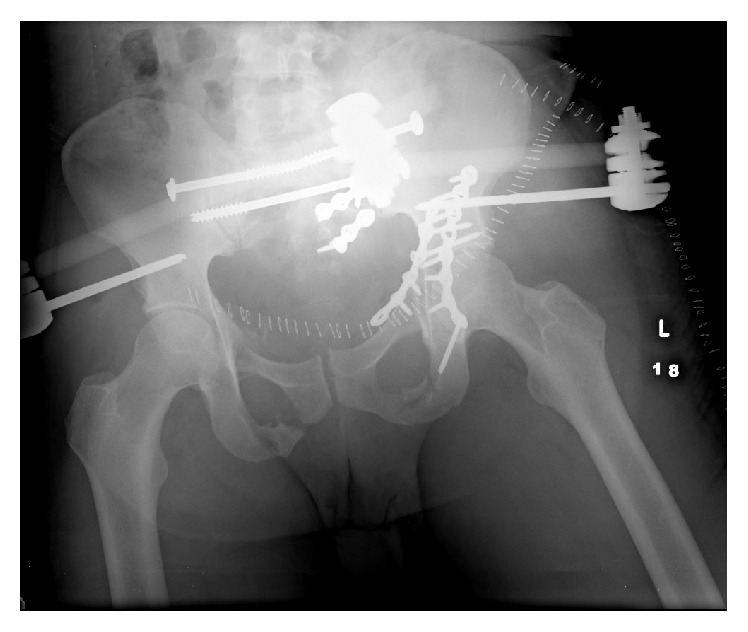
AP pelvis radiograph status after stabilization of the pelvis.

Subsequently, the patient returned to the operating room on hospital day six for further stabilization of the pelvis (Figure 5). The patient underwent open reduction of the left sacral fracture, percutaneous screw fixation of the posterior pelvic ring (right sacroiliac joint and left zone 2 sacral fracture), and stabilization of the anterior pelvic ring with anterior external fixation.

At the twelfth week of follow-up, the patient had no urologic or sexual dysfunction. His left-sided foot drop improved. At one year of follow-up, the patient is ambulating with a cane.

## 3. Discussion

As noted above, testicular dislocation often presents with a wide variety of traumatic injuries, most frequently due to a motorcycle collision, and it can easily be overlooked due to the severity of other injuries [2]. Testicular dislocation occurs when an upward force is applied directly to the scrotum, forcing either one or both testicles into the surrounding tissues [2, 3]. The most common region to be dislocated to is the superficial inguinal area [2, 9], and dislocations to the deep inguinal canal and the abdominal cavity have been reported as well [2, 10]. In cases associated with motorcycle collisions, the force to the scrotum is likely caused by the gasoline tank striking the rider's perineum and scrotal region due to rapid deceleration of the vehicle [3]. In the above case, the testicular dislocation in the presence of a lateral compression pelvic ring injury is suggestive of two separate traumas, as a testicular dislocation requires an upward force, and the lateral compression of the pelvic ring is the result of a laterally directed force. This insight can be useful in reconstructing the sequence of events of the motorcycle accident.

There are case reports describing testicular dislocation associated with anterior-posterior pelvic ring injuries and various types of non-pelvic ring-associated lesions, such as femoral, tibial, and foot fractures, as well as soft tissue injuries [1–5, 9–13]. Specifically, Boudissa et al. described a bilateral testicular dislocation presenting with a Tile B1 pelvic ring fracture [1] and Smith et al. reported a bilateral testicular dislocation presenting with a type II anterior-posterior compression pelvic ring injury [2]. Our case is particularly interesting as no other reports to our knowledge have presented a testicular dislocation with an associated lateral compression type injury of the pelvic ring or with an associated acetabulum fracture.

All motorcycle collision patients presenting with pelvic ring injuries or acetabulum fractures should be worked up for possible testicular dislocation with a scrotal exam. Pelvic ring injuries presenting with testicular dislocation should be managed with a urologic consultation, as the location of the dislocated testicle is intimately tied with the pelvic ring fixation surgical approach [1]. The initial diagnosis of testicular dislocation should be made during the patient's workup by physical exam. Direct palpation of the scrotum for the presence of two testicles will suffice [1, 2]. Any concern for a missing testicle should be confirmed with either ultrasound or CT [3–5].

Once the diagnosis is confirmed, surgical reduction and orchidopexy are required to prevent urologic and sexual sequelae, which include spermatogenesis, fertility, and endocrine issues [1, 2]. Open reduction is indicated when the testicle cannot be relocated via external manipulation or if testicular and/or spermatic cord integrity is in doubt [1].

For our patient, the Pfannenstiel approach was used to stabilize the anterior column of the acetabulum fracture [8]. As the most common location of the dislocated testicle is the superficial inguinal region [8], which is directly in the path of the Pfannenstiel approach, the unaware surgeon performing this procedure may risk causing iatrogenic injuries to the dislocated testicle [1].

Patients with pelvic ring injuries or testicular dislocations should be examined for signs of additional urological injuries, such as bleeding at the urethral meatus, a high riding prostate, or hematuria [14]. Studies have demonstrated that urogenital injuries may be present in 12–20% of patients with pelvic ring fractures and that there is a higher incidence in males [14, 15]. Posterior urethral tears and bladder rupture are the most common urogenital injuries associated with pelvic ring fractures [15]. Diagnosis is made with a retrograde urethrocystogram. Patients may require a temporary suprapubic catheter. Urological repairs should be done concomitantly with anterior ring stabilization to reduce the risk of infection [14]. Complications include urethral stricture, impotence, infection, anterior pelvic ring nonunion, and urinary incontinence [15].
